# Research on the factors influencing the process of prefabricated fragments penetrating finite thickness concrete

**DOI:** 10.1038/s41598-024-56024-2

**Published:** 2024-03-05

**Authors:** Zhenning Wang, Qingxin Li, Jianping Yin, Zhijun Wang, Xudong Li, Jianya Yi

**Affiliations:** 1 College of Mechanical and Electrical Engineering, North University of China, Taiyuan, 030051 China; 2 NORINCO GROUP Aviation Ammunition Institute, No.65, Nanzhi Road, Xiangfang District, Harbin City, 150036 China

**Keywords:** Prefabricated spherical fragments, Limited thickness concrete, Target back collapse, Motion state parameters, Engineering, Physics

## Abstract

The damage to the back of the target plate is a phenomenon that occurs when concrete is subjected to high-speed impact. In order to study the motion parameters of prefabricated spherical fragments penetrating finite thickness concrete targets at high speeds and the occurrence rules of concrete damage, as well as the impact of target back damage on the motion of fragments, experiments were conducted on 100 mm finite thickness concrete targets with prefabricated spherical fragments. The concrete model parameters in LS-DYNA were modified based on the residual velocity of fragments, and numerical simulations were conducted on the penetration of prefabricated fragments with different impact velocities and concrete target plates with different thicknesses. By analyzing the location of concrete target plate damage, the relationship between concrete thickness and concrete damage was obtained; Combining the motion parameters of fragment penetration process, the phenomenon of concrete collapse was linked to fragment motion, and the influence of concrete thickness on fragment motion parameters was analyzed. The results indicate that the thickness of the finite thickness concrete target plate and the penetration speed of fragments have a significant impact on the damage state of the target back, and further affect the motion change response stage during the penetration process of prefabricated fragments.

## Introduction

Concrete is composed of various materials such as water, cement, sand, and stones in a certain proportion. It is widely used in the construction of modern buildings, defense structures, and other structures. Concrete can change its strength according to different needs and be poured into different shapes and thicknesses according to needs, making it widely applicable and able to quickly complete the construction of buildings. In response to the widespread use of concrete buildings, the research on the endpoint effect of projectile penetration into concrete targets has increasingly become a concern in the field of weapon technology research and engineering protection^1^. In the last century, research on concrete penetration mainly focused on the penetration depth of concrete, and based on a large number of experiments, various empirical formulas were established to calculate the penetration depth of projectiles under different conditions. Among them, the more representative ones are the formulas proposed by Berezin, NDRC, Forrestal, and others^2–4^, which provide a reliable basis for the design of concrete protection.

The dynamic problems in the process of penetrating or impacting concrete are more complex, involving the target material, structural parameter characteristics and failure modes^5–9^. At present, the research methods of projectile penetrating concrete target mainly include experimental research, establishment of engineering model, correction of empirical formula, etc. With the continuous development of computer technology, numerical simulation and numerical calculation of penetration process have been relatively mature, and provide research means for detailed calculation and verification of experimental design^10^. Compared with the experiment, numerical simulation can combine a variety of variables and methods to carry out more complex analysis and research, and use appropriate constitutive relations to obtain a more realistic calculation model. Compared with relatively few experimental and theoretical studies, a large number of scholars have carried out numerical simulation work in the field of penetration and penetration of concrete^11–13^.

With the continuous improvement of protective requirements, the strength of concrete also continues to improve. Chen Xingming et al.^14^ analyzed the applicability of the empirical formula for penetration depth to high-strength concrete penetration, and conducted research and analysis on relevant experiments; Zhang Xueyan et al.^15^ compared the penetration tests of two different strength concrete types, C35 and C60, and analyzed the penetration depth of the projectile in the tests. They found that high-strength concrete not only improved its penetration resistance, but also had greater damage to the surface of the target plate; On the basis of these studies, Lv Yingqing et al.^16^ studied the mechanism of high-speed projectile penetration into ultra-high performance concrete, analyzed the penetration depth of the projectile in the experiment, and discussed the influence of steel fibers on the penetration results. In order to study the complex initial conditions of the projectile in practical engineering, Liu Tiansong et al.^17^ studied the influence of various variables on ballistic deflection during the oblique penetration of the projectile into concrete. ABAQUS was used to load the projectile response force function onto the surface of the projectile, and the centroid motion trajectory during the projectile penetration process was obtained; On the basis of plain concrete, Wu Haijun et al.^18^ considered the influence of steel bars on trajectory and established a trajectory calculation model that penetrates finite thickness concrete targets through the analysis of collision effects; Zhang Binwei et al.^19^ drew inspiration from the YOUNG formula and studied the calculation method of residual velocity for projectile penetration into multi-layer interval concrete targets.

In order to gain a deeper understanding of the failure mechanism of the medium during the process of projectile penetration into concrete, many scholars have discussed and studied algorithms and models during the penetration process based on cavity expansion theory and related engineering theories. Xue Jianfeng et al.^20^ studied the endpoint trajectory of oblique penetration and established a model based on the spalling mechanism that can predict the depth of penetration; Liu Zhilin et al.^21^ considered the free surface effect on the concrete target back, modified the function of projectile penetration resistance, and established an engineering calculation model that can predict various physical quantities such as penetration overload; Peng Yong et al.^1^ established an acceleration time history calculation model for projectile penetration into the target based on the dynamic spherical cavity expansion theory and considering various resistance function theoretical formulas. In terms of studying the endpoint effect of fragment penetration into concrete, there have been relatively in-depth theoretical achievements in the pit formation effect of concrete targets. Liu Haipeng et al.^22^ conducted a phased analysis of the pit formation process of projectile penetration into concrete surfaces and divided it into four parts; Wang Jie et al.^23^ combined experiments and literature to conduct dimensional analysis on parameters such as pit diameter, and obtained a formula for predicting pit depth; Li Ming et al.^24^ considered factors such as impact velocity and steel reinforcement, and established a formula for calculating pit formation effect and energy consumption, providing reference for the design and engineering protection of penetrating projectiles.

In summary, although the penetration experiment of concrete has been studied in many aspects, most of them focus on positive penetration, and there are few theoretical and experimental studies on oblique penetration. It is generally believed that the larger the inclination angle of the projectile, the larger the damage range on the front of the target plate and the lateral deflection of the projectile, the smaller the penetration depth, and even the phenomenon of bouncing^25,26^. Most of the existing research on oblique penetration focuses on slender rod projectiles, and there are few studies on spherical projectiles. In addition, the concrete target plate for hitting the target is generally thicker, and the research direction is mainly to improve the penetration depth of the projectile to the concrete target plate. There are few studies on the damage effect of the small thickness target plate, and different sizes of projectiles will produce different force effects, size effects, and structural strength effects during the penetration movement. These factors will become an important influence on the change of concrete failure forms.

The analysis of the process of projectile penetrating concrete is very complicated, mainly because there are too many influencing factors in the process of penetrating concrete. In order to clarify the relationship between the target back damage and the penetration movement of small-sized projectiles, the penetration test of spherical prefabricated fragments of finite-thick concrete was carried out in this paper. The numerical calculation model is used to analyze and discuss the influencing factors of the target back damage effect during the penetration process, and the movement process of the fragment is studied in combination with its failure form.

## Fragment penetration test of finite thickness concrete

**Figure 1:**
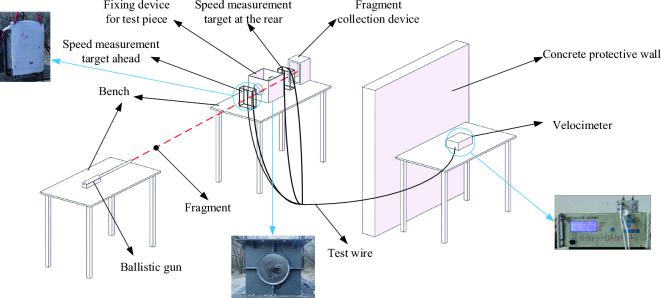
Schematic diagram of experimental testing plan.

**Figure 2:**
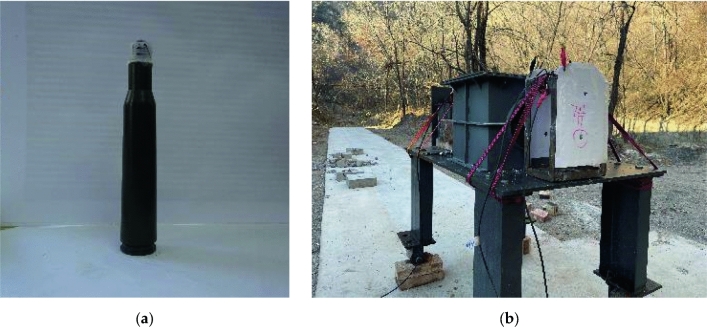
Physical diagram of the experimental testing device. (**a**) Is experimental cartridge (sabot, fragment); (**b**) is the experimental station.

In order to obtain the motion parameters of fragments penetrating a finite thickness concrete target plate and the damage state of concrete, penetration tests were conducted on fragments with diameters of 6 mm and 11 mm. Ballistic guns were used to launch a sabot equipped with prefabricated spherical fragments, allowing the fragments to pass through the velocity target paper located in the front of the concrete target plate, the concrete target block, the velocity target paper located in the rear of the concrete target plate, and the collection device. The testing method of the velocity measurement target paper is "on off" velocity measurement, and the four target papers are respectively connected to the six channel velocity meter by wires, enabling them to measure the initial velocity and remaining velocity of the prefabricated fragments. The testing plan for the experiment is shown in Figs. 1 and 2.

In Fig. 2a, from top to bottom, there are prefabricated spherical fragments, sabots, and propellant cartridges; In Fig. 2b, from right to left are the front velocity target, the target box with concrete target blocks, the rear velocity target, and the collection box.

The experimental platform is mainly composed of three parts: launcher, specimen fixing device and speed measuring device. The launching device is equipped with a 12.7 mm launching gun fixed on a steel launch pad as a carrier to launch a bullet with a prefabricated tungsten alloy spherical fragment at the front end. Since the spherical fragment cannot be directly fixed on the head of the cartridge case, it is fixed with a plastic sabot. The plastic sabot can play a role in closing the launching gas, so that the launching gas can efficiently promote the movement of the fragment. The change of the projectile fragment launch velocity is controlled by adjusting the quality of the propellant in the cartridge case, so that the projectile fragment can reach hundreds of initial velocities that can be changed.

As a target for high-speed impact with prefabricated fragments, the concrete specimen is fixed in an experimental box of a cube structure, which is made of welded steel plates. The top of the square box is open, and the target plate can be easily replaced. There are circular holes with D = 15 cm in the front and rear of the box, so that the high-speed moving fragments can directly hit the target plate. The lower part of the box is directly fixed with bolts and nuts; after loading the concrete specimen, the upper part of the box is also covered with a steel plate and fixed with bolts, which can ensure the safety of the experiment under high-speed impact and avoid the movement of the specimen and the box. A fixed steel box is placed behind the square box to catch the fragments passing through the concrete specimen. The size of the concrete specimen is 300 mm × 300 mm × 100 mm.

The speed measuring device is mainly composed of speed measuring target paper, speed measuring target frame, six-channel velocimeter and wire. A speed target frame is placed in the front and rear of the box where the test piece is placed, and a speed target paper is pasted in the front and rear of the frame. The two ends of each target paper are connected to the wire, and the other end of the wire is connected to the six-channel velocimeter. The working principle of the velocimeter is: determine the distance between the two target papers and input; in the experiment, the circuit is connected, and the circuit is short-circuited when the fragment penetrates the target paper, so the time interval through the two target papers can be measured in turn, so as to output the velocity value of the fragment.

The concrete specimen is placed in the square box, the box body and the cover plate are fixed, the speed measuring target paper is connected and placed, and the speed measuring instrument is connected; the projectile body (fragment, shell, charge) is filled in the launcher. After preparation, the fragments are fired, the initial velocity of the fragments and the residual velocity after passing through the concrete specimens are measured, and the failure state of the concrete specimens is checked.

The concrete material is mainly composed of ordinary Portland cement, quartz sand, aggregate and water, and the ratio is: 1:1.6:2.7:0.46. Among them, cement is the cementitious material of concrete, which plays a bonding role; sand is used to fill the gap between cement and aggregate; aggregate plays a role in enhancing the mechanical properties of concrete; water is the role of regulating fluidity. Firstly, the weighed quartz sand is poured into the mixer for stirring. After it is stirred evenly, the weighed cement and aggregate are poured into the mixer for further stirring. Finally, the water and water reducing agent are weighed and poured into the mixer to form a slurry with good fluidity. The prepared slurry was poured into a square mold of 300 mm × 300 mm × 100 mm, and finally the surface of the specimen was flattened. After 30 days of curing, the specimens were taken out of the mold, and the average axial compressive strength was measured to be 35 MPa.

In the experiment, the initial velocity of the prefabricated fragments was changed by changing the amount of propellant in the cartridge. A total of 9 experiments were conducted, and the experimental conditions are shown in Table 1.

**Table 1:** Test Conditions for Fragment Penetration into Concrete.

NO	Diameter (mm)	Target	Velocity before target (m/s)	Velocity behind target (m/s)	Penetration or not
1	6	Concrete	783	0	Not
2	6	Concrete	860	0	Not
3	6	Concrete	926	0	Not
4	6	Concrete	940	0	Not
5	6	Concrete	1142	0	Not
6	6	Concrete	1486	0	Not
7	11	Concrete	903	26.59	Penetration
8	11	Concrete	983	83.57	Penetration
9	11	Concrete	1177	234.17	Penetration

**Figure 3:**
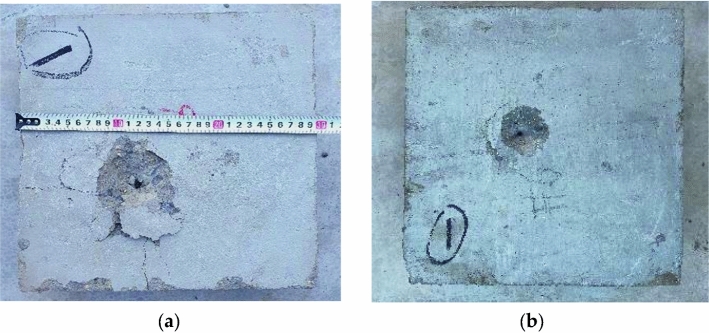
Failure state of 6 mm fragments on concrete. (**a**) Is 1142 m/s; (**b**) is 783 m/s.

**Figure 4:**
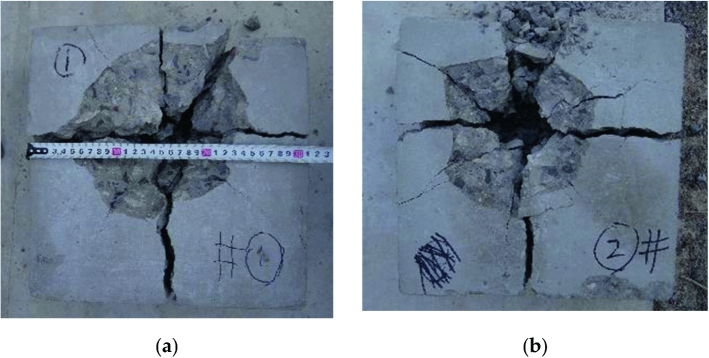
Failure state of 11 mm fragments on concrete. (**a**) Is 903 m/s; (**b**) is 983 m/s.

From Table 1, it can be seen that within the experimental fragment velocity range, 6 mm prefabricated fragments are difficult to penetrate 100 mm thick concrete target plates, while 11 mm fragments can easily penetrate target plates. The destructive effect of fragments penetrating concrete is shown in Figs. 3 and 4.

From Figs. 3 and 4, it can be seen that when the fragments do not penetrate the concrete target plate, the failure form of the concrete target plate is surface pit formation + tunnel; When penetrating the concrete target plate, the failure form of the concrete target plate is surface pit + tunnel + back pit. As the initial velocity of the fragments increases, the penetration depth of 6 mm fragments continues to increase, while the remaining velocity of 11 mm fragments continues to increase.

## Numerical verification and calculation of fragment penetrating concrete

In order to conduct in-depth research on the back target collapse effect of prefabricated spherical fragments and the motion state of prefabricated fragments, it is necessary to establish a model of the process of fragments penetrating concrete based on experimental results, and use the numerical model in LS-DYNA as the basis for calculation to meet the numerical calculation of penetration under various working conditions. Based on the actual situation of the two materials, the Lagrange contact method was selected between the fragments and concrete. The JC model was selected for the prefabricated tungsten alloy spherical fragments, and the RHT model was selected for the concrete. The strength of the concrete was 35 MPa, and the model parameters of the two materials are shown in Tables 2 and 3.

**Table 2:** Main Model Parameters of Tungsten Alloy Materials (unit: mm; ms; kg).

*R*_0_	*G*	*E*	*PR*	*A*	*B*	*N*	*C*	*M*	*TM*	*TR*	*EPSO*
1.75e^-5^	137	350	0.22	1.51	0.177	0.12	0.016	1.0	1498	294	0.001

**Table 3:** Main Model Parameters of Concrete Materials(unit: mm; ms; kg).

*R*_0_	*SHEAR*	*EPSF*	*B*_0_	*B*_1_	*T*_1_	*FC*	*FS*	*FT*	*Q*_0_	*B*	*T*_2_
2.7e^-6^	26.7	2	1.22	1.22	100	0.035	0.18	0.1	0.6805	0.0105	0.0

In Table 2, R0 represents the material density; *G* is the shear modulus; *E* is the elastic modulus; *PR* is Poisson's ratio; *A*, *B*, *N*, *C*, and *M* are the parameters of the relevant equation; *TM* is the melting temperature; *TR* is room temperature; *EPSO* is the effective plastic strain rate. In Table 3, R0 represents the material density; *SHEAR* is the elastic shear modulus; *EPSF* represents erosion plastic strain; *B*_0_, *B*_1_, *T*_1_, and *T*_2_ are polynomial *EOS* parameters; *FC* is the compressive strength; *FS* is the relative shear strength; *FT* is the relative tensile strength; *Q*_0_ and *B* are the Lode angle correlation coefficients.

The RHT dynamic constitutive model has been widely used in structural impact analysis because it can describe the unrecoverable strain of materials during explosion and reflect the damage evolution characteristics of concrete. The model includes pressure-dependent elastic limit surface, failure surface and residual strength surface, which can describe the variation of initial yield strength, failure strength and residual strength of materials.

In order to better simulate the real experimental conditions, the numerical model uses a full-scale calculation model. The mesh size of the target plate is 1.5 mm, and the regular hexahedral mesh is used. The mesh size of the spherical fragment is 1.5 mm, and the butterfly mesh is used. The contact between the target plate and the projectile body adopts surface erosion contact. In LS-DYNA, the keyword * CONTACT_EROSION_SURFACE_TO_SURFACE is used for definition. The projectile is set as the active surface and the target plate is set as the passive surface. Since the RHT model does not define the parameters of failure deletion, in order to avoid the calculation overflow at high loading rate, the keyword * MAT_ADD_EROSION is added to delete the elements with large deformation.

Based on the damage results of prefabricated fragments on concrete target plates in the experiment, including parameters such as residual velocity and penetration depth, the numerical calculation model was validated. The comparison between the numerical calculation results and experimental results is shown in Table 4:

**Table 4:** Comparison of parameters between numerical simulation and experimental results.

No	Velocity before target	Residual velocity/penetration depth	Numerical simulation results	Relative error
1	783 m/s	32 mm	31 mm	3.13%
2	860 m/s	28 mm	35 mm	25%
3	926 m/s	34 mm	39 mm	14.71%
4	940 m/s	31 mm	38.5 mm	24.19%
5	1142 m/s	53 mm	44.8 mm	15.47%
6	1486 m/s	57 mm	52.2 mm	8.42%
7	903 m/s	26.59 m/s	21.31 m/s	19.85%
8	983 m/s	83.57 m/s	59.22 m/s	29.14%
9	1177 m/s	234.17 m/s	187.3 m/s	20.02%

From Table 4, it can be seen that the average error of the depth of penetration of 6 mm fragments into concrete slabs is 15.15%, and the average error of the remaining velocity after penetration of 11 mm fragments into concrete is 23%. Combined with the damage combination of concrete in numerical simulation, it can be seen that the damage mode calculated by numerical calculation is the same as the experimental results, with good consistency.

On this basis, the remaining velocity calculation formula for the penetration of prefabricated tungsten alloy fragments into finite thickness concrete targets is first obtained. The experimental results are the main focus, and some numerical simulation results are added to make the fitting formula more accurate. The numerical calculation scheme for the remaining velocity of fragments includes prefabricated fragments with initial velocities ranging from 1200 to 1500 m/s, with velocity intervals of 50 m/s, totaling 7 groups.

In order to study the effect of different thicknesses of finite thickness concrete on the penetration process of prefabricated fragments, 50–100 mm thick concrete was selected as the target plate, with a gradient of 10 mm between thicknesses. Fragments with different velocity gradients were used to penetrate the target plate, with a velocity of 500–1500 m/s and a gradient of 500 m/s. The numerical simulation scheme for the penetration of prefabricated fragments into concrete targets of different thicknesses is shown in Table 5.

**Table 5:** Numerical Simulation Scheme for Prefabricated Fragments Penetrating Concrete Target Plates of Different thicknesses.

Variable type	Diameter	Initial speed	Concrete thickness
Level	10 mm	500m/s 1000 m/s 1500 m/s	30 mm 40 mm 50 mm 60 mm 70 mm 80 mm 90 mm 100 mm

In addition, in practical situations, it is generally impossible for fragments to penetrate the surface of concrete targets vertically. In this case, it is necessary to consider the impact of the angle of attack of fragments on the penetration process, as well as the damage effect on concrete during oblique penetration and the motion parameters of fragments. Based on this, taking 1500 m/s fragments as an example, a total of 18 sets of simulations are conducted with the thickness of concrete and the angle of attack of fragments as independent variables, starting from 0° to 85° and at intervals of 5°.

## Effect of target back damage on fragment motion parameters

### Calculation of prefabricated fragment remaining based on poncelet formula

The Poncelet formula is a calculation method used to describe the penetration of a projectile into a solid medium, and has been widely used in the calculation of solid materials such as concrete. The formula for calculating the remaining velocity of the Poncelet formula is shown below. It can be seen that it takes into account many parameters such as the shape of the projectile, as well as parameters such as the density and thickness of the target plate. Since the penetration body in this article is a spherical fragment, formula (1) can be simplified to the form of formula (2). Table 6 is the data used in the residual velocity formula after the fragment penetrates the 100 mm concrete target plate, which includes the filtered experimental data and the data obtained by simulation calculation.

1 $$ v^{2} = v_{0}^{2} \exp ( - \frac{{2c_{3} Ah}}{m}) + \frac{{c_{1} }}{{c_{3} }}\exp ( - \frac{{2c_{3} Ah}}{m}) + 1 $$

2 $$ v^{2} = v_{0}^{2} \exp ( - \frac{{c_{3} h}}{r\rho }) + \frac{{c_{1} }}{{c_{3} }}\exp ( - \frac{{c_{3} h}}{r\rho }) + 1 $$

**Table 6:** Numerical Simulation Calculation and Experimental Data of Fragment Residual Velocity.

No	Initial speed (m/s)	Residual velocity (m/s)	Notes
1	903	26.59	Experimental data
2	983	83.57	Experimental data
3	1177	234.17	Experimental data
4	1200	206.0	Simulation data
5	1250	229.7	Simulation data
6	1300	272.1	Simulation data
7	1350	301.5	Simulation data
8	1400	330.7	Simulation data
9	1450	365.5	Simulation data
10	1500	391.6	Simulation data

Because there are only three groups of experiments penetrating 100 mm concrete, and the velocity distribution range is only 903–1177 m/s, the numerical simulation results of 1200–1500 m/s are added as auxiliary fitting on this basis. Due to the lack of experimental data, the fitting results are only used as a reference form in this velocity range, and the experimental results need to be added for further optimization. As a linear fitting of v2 and v02. According to the fitting results, the values of unknown parameters c1 and c3 in the formula can be obtained. Therefore, the formula for calculating the residual velocity of fragment penetrating finite-thick concrete is shown in (3):

3 $$ v^{2} = v_{0}^{2} \exp ( - \frac{2155.6h}{{r\rho }}) + \frac{94753180.1}{{2155.6}}\exp ( - \frac{2155.6h}{{r\rho }}) + 1 $$

### The influence of concrete thickness on the penetration process

**Figure 5:**
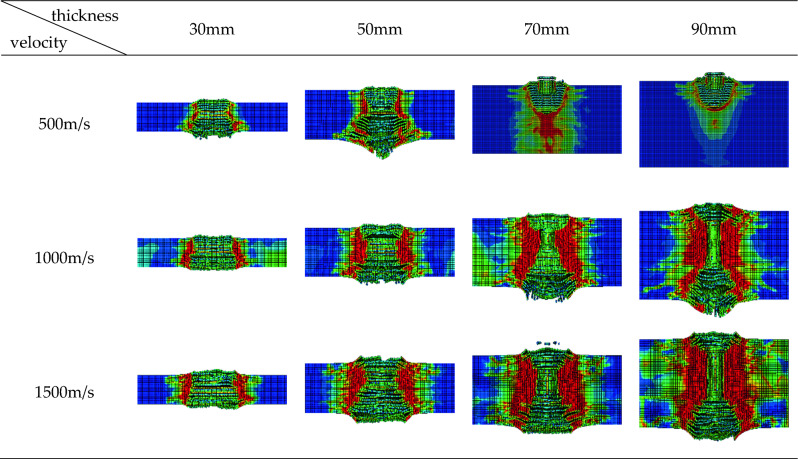
Different thicknesses lead to different forms of concrete damage.

The thickness of finite thickness concrete has a significant impact on the penetration process of fragments. This is mainly because the thickness of concrete directly affects the length of the penetration path, resulting in differences in the damage stages of concrete in the direction of fragment penetration, leading to a re division of the scope of the three areas: excavation area, tunnel area, and collapse area, as shown in Fig. 5. The thickness of concrete can affect the resistance and motion state of fragments during penetration, leading to irregular changes in the remaining velocity of fragments.

From Fig. 5, it can be seen that as the thickness of the concrete target plate increases, the division of various areas of concrete at different speeds gradually becomes clearer. There are three obvious areas starting from 70 mm, and the opening area of concrete with different thicknesses has slightly decreased; The tunnel area gradually lengthens; There is also a slight decrease in the collapse area.

In order to study the effect of concrete thickness on fragment motion, the numerical simulation results in Table 5 were used to analyze the degree to which the thickness of concrete affects the resistance of fragments during penetration. Figure 5 shows the force acting on fragments during penetration into concrete target plates.

Although the force on fragments is prone to fluctuations due to fluctuations in their motion state, there are still traces of the variation in resistance. The penetration resistance of fragments with a speed of 500 m/s remains basically consistent between 0.005 and 0.015 ms, and reaches its maximum resistance value around 0.025 ms. This can be regarded as the maximum force moment when the fragments fully contact the concrete target during penetration and the velocity is high. Afterwards, influenced by the thickness of the target plate, the force duration of the fragments increases with the increase of the thickness of the target plate, and the integral of their resistance value increases with time. The 1000 m/s and 1500 m/s have the same variation pattern during the penetration process. As the initial velocity increases, the moment when the fragments are subjected to the maximum resistance gradually decreases.

**Figure 6:**
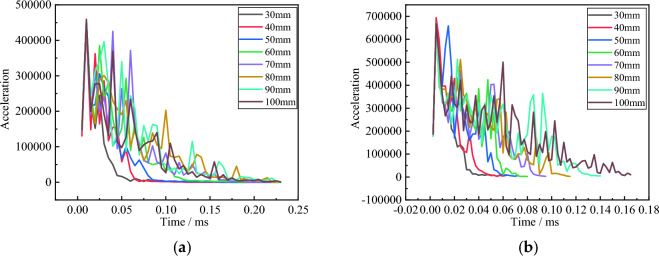
Variation of fragment acceleration with time at 1000 m/s and 1500 m/s. (**a**) Is 1000 m/s; (**b**) is 1500 m/s.

**Figure 7:**
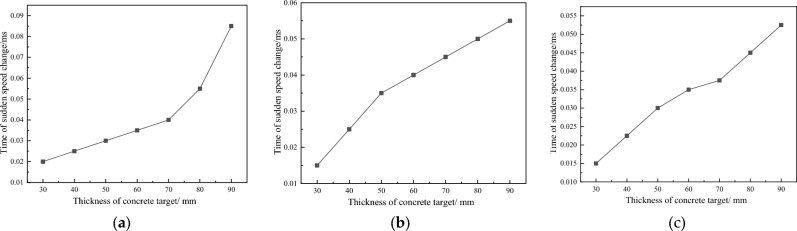
Changes in the Time of Sudden Speed Change with Concrete Thickness. (**a**) Is 500 m/s; (**b**) is 1000 m/s; (**c**) is 1500 m/s.

From Fig. 6, it can be seen that the penetration resistance of fragments at the same speed during excavation is basically the same. As time goes on, compared to infinitely thick concrete, the resistance of fragments in the control group with smaller thickness begins to decrease, resulting in a velocity reversal at a certain moment. This is the moment when the velocity undergoes a sudden change, and the velocity of concrete with different thicknesses changes as shown in Fig. 7.

From Fig. 7, it can be seen that with the increase of concrete thickness, the sudden change in fragment velocity shows a linear growth trend. As the fragment velocity increases, the occurrence time of the response time gradually decreases. Figure 7 shows the variation of residual velocity of concrete fragments with different thicknesses over time under three different velocity gradients.

**Figure 8:**
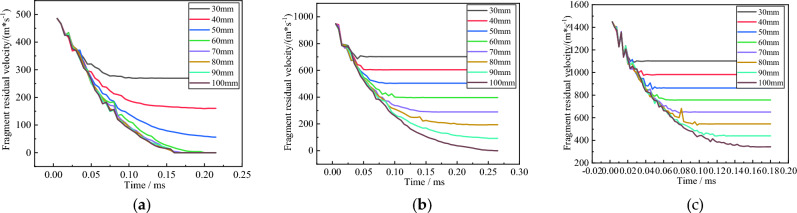
Time dependent variation of residual velocity of fragments of concrete with different thicknesses. (**a**) Is 500 m/s; (**b**) is 1000 m/s; (**c**) is 1500 m/s.

From Fig. 8, it can be seen that the larger the concrete thickness, the smaller the residual velocity of fragments, and the effect of concrete thickness on the residual velocity of fragments shows a linear decreasing trend. As the speed of fragments increases, the process of changing the motion state between fragments becomes more apparent. During the movement of fragments, the damage state of the target back has a direct impact on the movement process of fragments. Due to the situation where fragments do not penetrate the target plate at a speed of 500 m/s, only the damage situation of target plates at 1000 m/s and 1500 m/s is currently analyzed. Figure 8 shows the relationship between the occurrence time of damage to the back plate concrete and the thickness of the concrete target plate under two different speeds.

**Figure 9:**
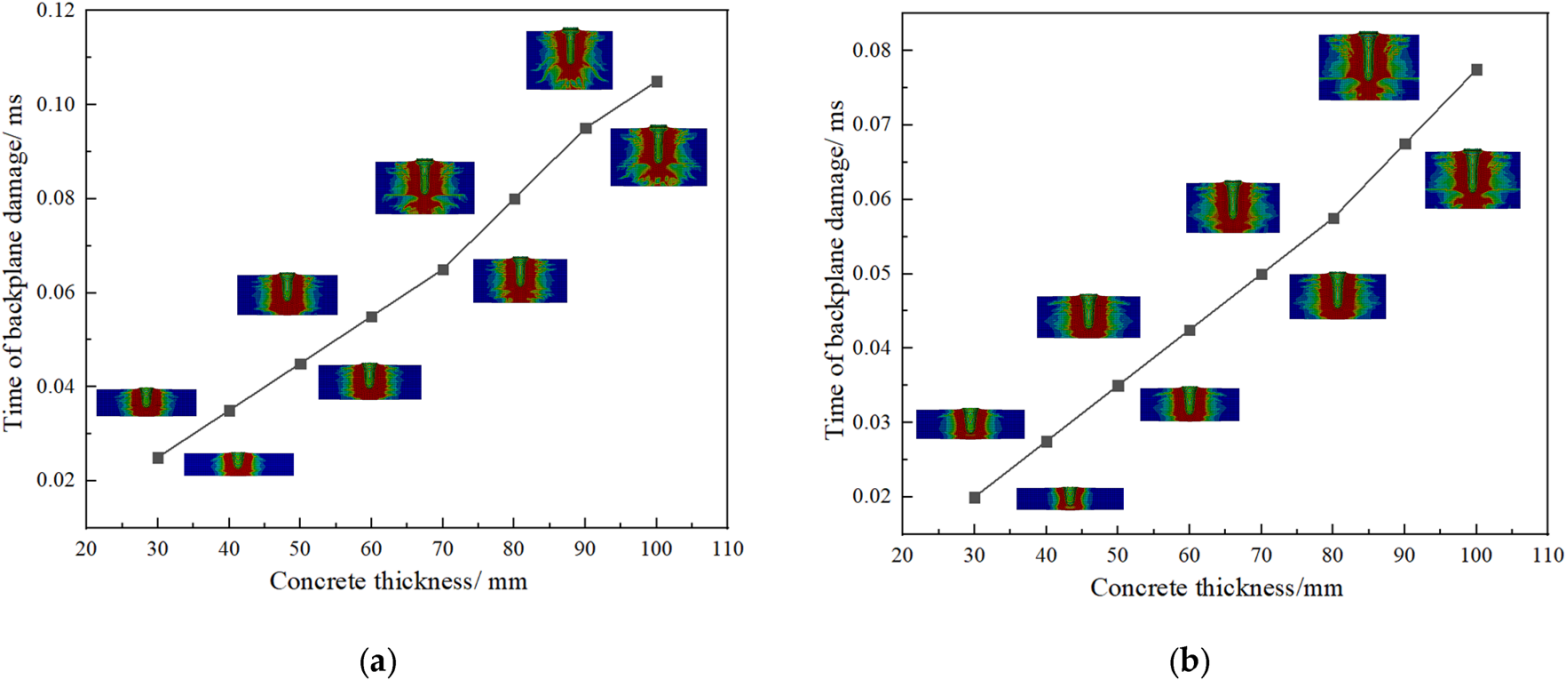
Changes in the Time of Damage to Concrete Backboard with Concrete Thickness. (**a**) Is 1000 m/s; (**b**) is 1500 m/s.

Based on the previous research, the penetration process of 100 mm thick concrete was studied. Figure 9 shows the velocity and acceleration variation curve of a 1000 m/s fragment penetrating the concrete, and the penetration status positions (pit opening, tunnel, back plate collapse) and concrete damage maps of each node change were attached.

4 $$ a = \frac{300061.7}{{1 + \left( {\frac{t}{0.06829}} \right)^{2.12284} }} - 24066.9 $$

**Figure 10:**
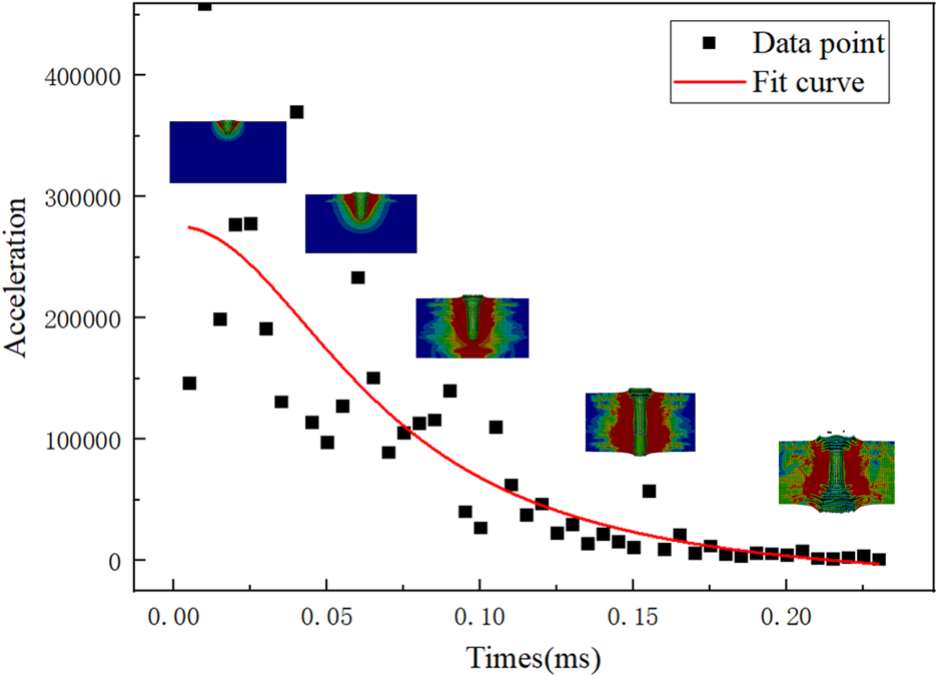
Fitting Curve of Stress on 100 mm Concrete Fragments at 1000 m/s.

**Figure 11:**
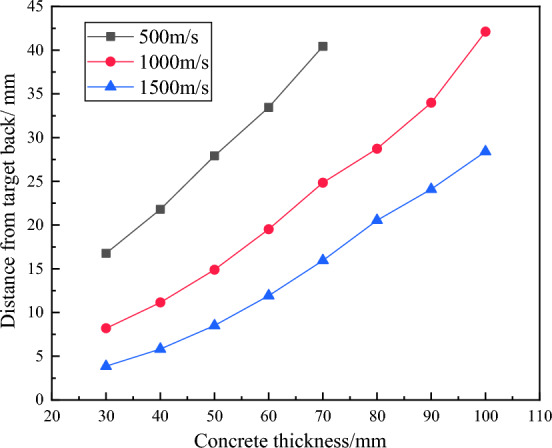
Distance from the location of fragment backboard collapse to the target back.

From Fig. 10, it can be seen that the motion state of fragments can be roughly divided into three stages: the penetration resistance index increases when the fragments open the pit, the resistance changes uniformly when entering the tunnel area, and the penetration resistance gradually decreases when the back target cracks. Based on this feature, as the thickness of the target plate changes, the position where the fragments undergo acceleration changes. The distance from the position where the fragments undergo acceleration changes to the back of the target under different concrete thicknesses is shown in Fig. 11.

It can be seen from Fig. 11 that with the increase of fragment velocity, the damage area at the back of the fragment target changes. The distance from the damage area to the back of the target increases with the increase of concrete thickness, and the increase of fragment velocity leads to the lag of the damage area and the distance from the back of the target decreases. The change of concrete thickness leads to the change of back target damage area and further affects the motion parameters of fragments. At this time, it is necessary to modify the calculation formula of fragment residual velocity according to different concrete thickness to make the calculation result more accurate.

### Impact of fragment attack angle on penetration process

**Figure 12:**
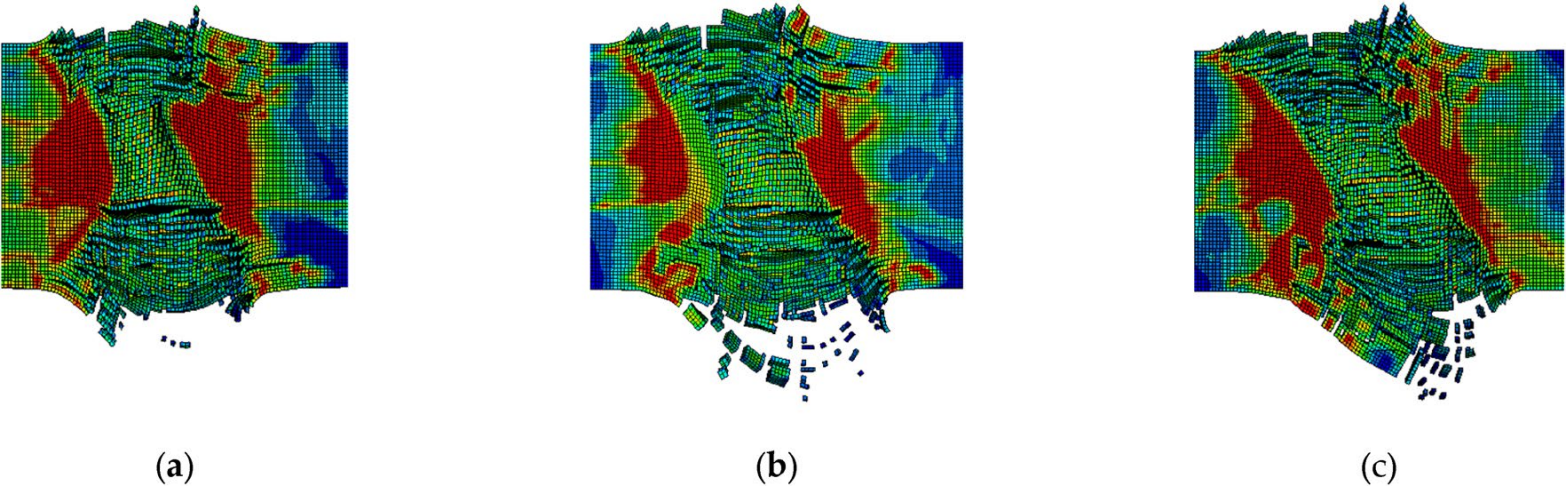
Differences in Concrete Damage States at Different Angles of Attack. (**a**) Is 20°; (**b**) is 30°; (**c**) is 40°.

When there is an angle of attack at the initial velocity of the fragments, that is, when the fragments penetrate obliquely, the vertical velocity of the fragments will be greatly reduced, resulting in the fragments being unable to penetrate a finite thickness concrete target plate and affecting the trajectory of the fragments^27^. At the same time, the oblique penetration process of fragments will significantly increase the contact path with concrete and change the damage state of the concrete target plate. When the penetration angle is constant, as the thickness of the concrete increases, the remaining velocity and penetration depth of the fragments continue to decrease, and different penetration areas are formed in the concrete. Taking 20°, 30°, and 40° as examples, the comparison of concrete damage at different attack angles is shown in Fig. 12.

**Figure 13:**
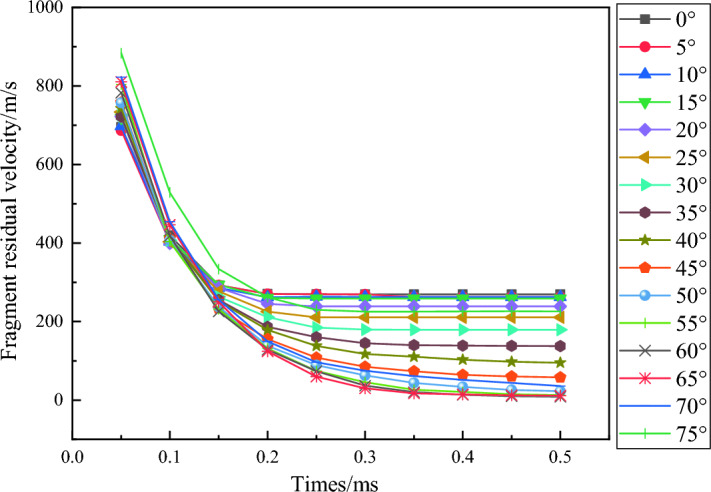
Relationship between fragment velocity and time at different angles of attack.

When the thickness of the concrete is constant and the penetration angle is different, the impact of the attack angle of the fragments on the damage area of the concrete is most obvious. In Fig. 12, as the attack angle of the fragments increases, the area of the concrete surface opening area gradually increases, and the target back collapse area also gradually increases. The existence of the angle of attack directly affects the penetration depth of fragments in the vertical direction of concrete, leading to a longer penetration trajectory and longer penetration resistance action time. The time curve of the penetration speed of fragments at different angles of attack over time is shown in Fig. 13.

**Figure 14:**
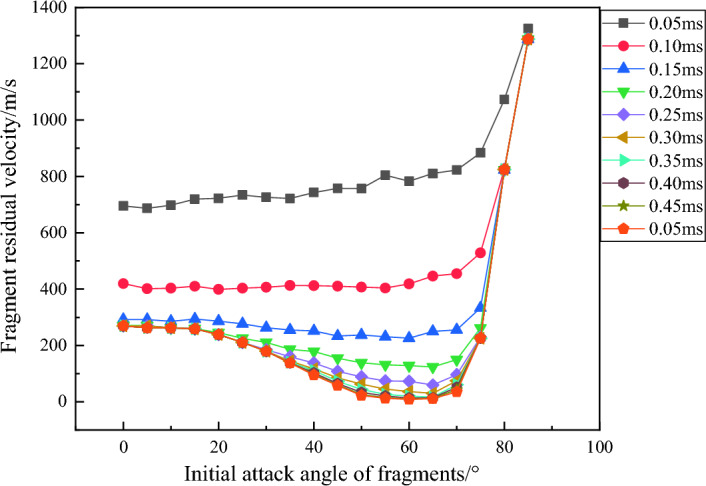
Relationship between fragment velocity and angle of attack at different times.

From Fig. 13, it can be seen that in the range of 0–60°, the remaining velocity of fragments decreases with the increase of attack angle, while in the range of 60–85°, the velocity increases due to the upward movement of fragment trajectory. The impact of the angle of attack on velocity exhibits bipolarity, which can be better reflected in the variation of the remaining velocity of fragments with the angle of attack at different times, as shown in Fig. 14.

**Figure 15:**
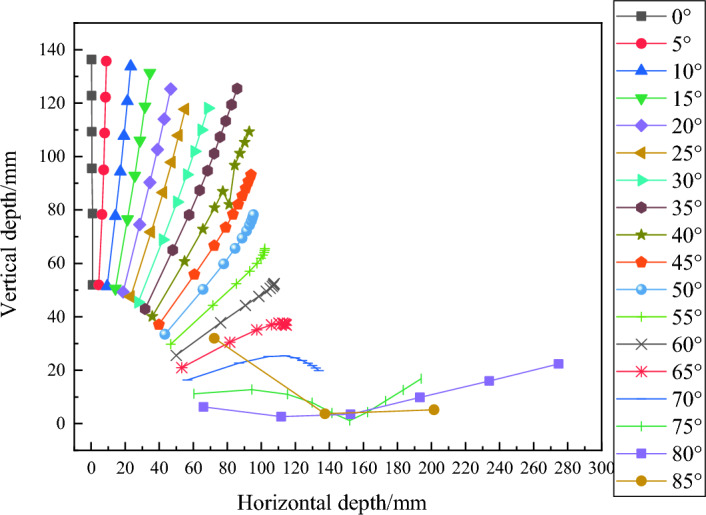
Fragment trajectory curves at different angles of attack.

Figure 15 shows the trajectory of fragments at various angles of attack when penetrating infinite thick concrete.

An interesting phenomenon can be observed from Fig. 15, where different deviations occur on the left and right sides near an attack angle of 50°. At 0–45°, the trajectory of the fragments deviates towards the vertical direction; At 55–85°, the fragments are inclined towards the horizontal direction. As the angle of attack increases, the impact on the resistance of fragment penetration is also greatest near 50°. Due to the same time interval between adjacent sample points, the variation trend of fragment velocity can be observed through the density of sample points. After 50°, fragments will gradually move towards the trend of bouncing. Considering the impact of attack angle, further research is needed on the calculation method of fragment residual velocity.

## Summary

This article verifies the numerical simulation model of concrete by conducting experiments on the penetration of prefabricated tungsten alloy fragments into finite thickness concrete. Based on the motion parameters of the fragments, the influence of different forms caused by concrete thickness on the penetration process of prefabricated fragments is analyzed. Combined with the relationship between the speed and acceleration of the fragments over time, the occurrence of damage in concrete is discovered. And a preliminary analysis was conducted on the impact of the attack angle of fragments on concrete damage, as well as the changes in fragment motion parameters and trajectories under different attack angles.

1. Based on the Poncelet formula, a formula for calculating the residual velocity of spherical fragments penetrating finite thickness concrete targets was obtained through numerical simulation and experimental results fitting.
2. On the basis of vertical penetration, the different forms of damage areas caused by concrete thickness were analyzed, and the location of the target back damage area was obtained by combining the fragment motion parameters. The law between the target back damage situation of different thicknesses of concrete and the fragment motion parameters was found.
3. The angle of attack of fragments directly affects the damage area of concrete, with different trajectory deviation patterns around 40°. The angle of attack causes changes in the damage area of the target back and changes the penetration resistance of fragments.

Combined with the selected concrete penetration model, it can be seen that the penetration process of finite thickness concrete is quite different from that of infinite thickness concrete, and the movement process of projectile and the failure process of concrete back are quite different. The motion state of small spherical projectile is affected by the thickness of the target plate and the initial velocity, which affects the residual velocity of the fragment. The oblique penetration process of spherical fragments and the deflection process of long rod projectiles show different rules, and the next step needs to be further explored in combination with experiments.

## Data Availability

The data presented in this study are available on request from the corresponding author. The data are not publicly available due to programming privacy in structural design.
